# Comparison of Medical Tape Performance Using Skin Response Quantitative Measurements on Healthy Volunteers

**DOI:** 10.7759/cureus.56548

**Published:** 2024-03-20

**Authors:** Michael J Turnbull, Iwen Grigsby, Karl Unertl, Kerry Sokol, Tera Nordby, Cedric Liu, Anna Bailey, Brian Spiewak, Graham Smith, Amy K McNulty

**Affiliations:** 1 Medical Solutions Division, 3M Health Care, St. Paul, USA; 2 Global Medical and Clinical Affairs, 3M Health Care, St. Paul, USA

**Keywords:** healthy human volunteers, medical tapes, clinical study, test methods, medical adhesive-related skin injury (marsi), skin integrity

## Abstract

Background: Medical tapes can lead to skin damage upon removal in susceptible patients with fragile skin and at higher risk of developing tissue injury.

Purpose: We compared the effect of medical tapes with silicone-based versus acrylate-based adhesives on the back or volar forearm stratum corneum using analytical techniques to assess skin condition and potential damage post product removal on 88 healthy volunteers.

Methods: Two studies were conducted in separate facilities (Study 1: 3M In-house Clinical Facility, St. Paul, Minnesota; Study 2: DermiCo, LLC, Broomall, Pennsylvania). Four commercially available tapes were the same in both studies, two for each type of adhesive. We evaluated adhesion to the skin, total proteins and corneocytes removed by the tapes, changes in transepidermal water loss (TEWL), and induction of the inflammatory cytokine interleukin-1 alpha (IL-1a).

Results: One of the silicone tapes displayed the strongest adhesion at 24 hours, and one of the acrylate tapes had the lowest adhesion, showing differences in performance within adhesive categories. The adhesion forces did not correlate with the amount of total protein or corneocytes removed. Silicone adhesives removed less total protein and corneocytes than acrylate adhesives. Silicone adhesives did not alter TEWL, whereas acrylate adhesives significantly raised TEWL. There were no differences in interleukin-1alpha induction.

Conclusion: The silicone adhesive tapes were less disruptive to the skin barrier than the acrylate adhesive tapes, even in healthy volunteers whose skin is not as fragile as what is observed in typical patients. This type of data could guide clinical product usage decisions.

## Introduction

Healthy skin plays a critical and complex role in the human body’s protection against damage and infection. Preserving the integrity of the skin barrier is a critical component of patient care that helps to mitigate the risk of infection from outside contaminants and microorganisms [1]; however, adhesives from medical devices can cause skin injuries during wear and removal. Healthcare-associated infections (HAIs) are a significant cost to the United States healthcare system [2], and reducing this economic burden can be accomplished, in part, through awareness and prevention of medical adhesive-related skin injuries (MARSIs), a term describing skin barrier damage associated with the wear and removal of adhesive-containing medical tapes, dressings, and drapes. MARSI awareness and prevention is especially critical in populations with fragile skin - infants, elderly, and those with chronic conditions, such as diabetes - and underscores the need to select the appropriate medical adhesives for patients at a higher risk of developing MARSIs [3,4].

Medical tapes with strong adhesive performance yet low MARSI risk are desirable to adhere to the skin with minimal impact on skin health during wear and removal. Various medical adhesive chemistries are available (acrylates, silicones, natural and synthetic rubbers, hydrocolloids, hydrogels, and polyurethanes) and impact the nature of the adhesion to the skin. The flow of the adhesive formulation into the crevices of the skin impacts the level of adhesion and therefore modulates the trauma upon removal [5,6]. The key is to select an adhesive with just enough bonding strength to achieve the clinical function needed.

To understand the extent to which medical adhesive products are linked to skin injury, several bioanalytical methods have been established to evaluate the disturbance of the outermost layer of the epidermis known as the stratum corneum (SC) [7-12]. Human SC is composed of layers of dead and cornified keratinocytes termed corneocytes. Such cornified layers provide the front-line protection against physical abrasions, chemical stimulants, and pathogens [13]. As the outermost layer of the skin, corneocytes are the first layer to lift off from adhesive device removal, and their presence on a device can be measured to assess how much skin is stripped by medical adhesives. Similarly, quantification of other biological substances, especially after repeated skin stripping, also serves as an indicator of the risk level of MARSIs. The outcomes of such measurement methods are often influenced by experimental variables, such as skin preparation, collection pressure and technique, and ambient conditions.

If the skin barrier is damaged, transepidermal water loss (TEWL) becomes elevated. International guidelines have been developed for measuring TEWL and skin hydration in non-clinical settings to assess workplace exposure to physical and chemical stressors [14]. This methodology has been used by others to examine the effect of the repetitive application and removal of various medical tapes and adhesive devices on skin integrity for the purpose of comparing adhesive product gentleness [8,15].

The purpose of this study was to quantitatively compare the effect of various medical tapes on the SC, using tapes with acrylate adhesives and tapes with silicone adhesives. Silicone-based adhesive medical products have been in the market for some time and are known to be less aggressive upon removal than acrylate-based adhesive counterparts [8,10,16]. However, this benefit often comes at a cost of lower adhesion performance. Our assessment included a newer silicone tape (designed to adhere strongly to skin with minimal skin health impact during wear and removal) alongside an established one, as we wanted to assess possible differences between silicone adhesives.

## Materials and methods

We devised a testing strategy incorporating several tests with the following rationale. Skin adhesion was tested to assess the performance of the tapes. We also assessed total protein removal by the tapes as a measurement of skin damage, which was confirmed by quantifying corneocytes removed. In addition, we measured interleukin-1a (IL-1a), which has been linked to many skin conditions, such as psoriasis and atopic dermatitis [17,18], as a marker of inflammation. Finally, since the SC is the main barrier to water evaporating through the epidermis to the external environment, we measured TEWL as it is accepted as a reliable marker for the skin barrier status. Two different studies were conducted in separate facilities. Each study tested eight (8) types of tapes, but only four of the eight tapes were common across both studies. These four tapes are the focus of this paper and are described in Table 1. The healthy volunteers were recruited with similar criteria for both studies (male or female over 18 and under 65 years of age with intact skin at applicable test sites; no psoriasis, eczema, or atopic dermatitis; no sunburns, tattoos, scars, cuts, acne or broken skin on the test sites; no sensitivity or allergies to acrylates or isocyanates; willingness to withhold topical products for at least 24 hours prior to sample application) and were compensated for their participation. Sample sizes were calculated based on results from a previous study to provide at least 80% power to detect a difference of 20% in adhesion (Study 1) or a difference of 1 g/m^2^*h in TEWL (Study 2).

**Table 1:** Tapes tested

Tape ID	Commercial name, Manufacturer	Type of medical adhesive	Type of backing
Tape A	3M™ Micropore™ S (3M Company, St. Paul, MN)	Silicone-based	Paper
Tape B	Leukoplast® Skin Sensitive Tape (BSN Medical, Hamburg, Germany)	Silicone-based	Paper
Tape C	Leukopor® paper (BSN Medical, Hamburg, Germany)	Acrylate-based	Paper
Tape D	Leukofix® (BSN Medical, Hamburg, Germany)	Acrylate-based	Plastic

Study 1

Study 1 (3M Center, St. Paul, MN) was a balanced, complete block design with eight (8) samples on the backs of 32 healthy human volunteers. The protocol was approved by our 3M Health Care Institutional Review Board, and informed consent was obtained. From this study, the endpoints of interest that allowed us to quantitatively compare the four tapes were skin adhesion and total protein removed by the tapes (bicinchoninic acid (BCA) method), analyzed at the 24-hour time point. Samples were applied in duplicate. Controls consisted of blank tape samples not applied to the skin and subjected to the same storage and bioassay conditions as the test samples.

Skin Preparation

The skin was washed with a 1% suspension of Neutrogena Ultra Gentle Daily Cleanser in water, rinsed, and patted dry.

Skin Adhesion Test Method

Tape strips (5 in length) were applied in duplicate to the back of the subjects and pressed into place with a 4.5-pound roller (once forward and back) to ensure uniform contact. The next day (t = 24 hours), the samples were observed and any lift was noted. Tape strips with >50% lift were manually removed and excluded from the analysis. An IMASS SP-2100 peel tester (IMASS Inc., Strongsville, OH) was used to remove the test samples using a controlled angle of 180 degrees and a peel rate of 90 inches per minute. Instantaneous peel force readings were recorded and averaged over the length of the peel. Collected tape strips were stored adhesive side up in sterile trays at -80°C until biological analysis.

Total Protein Method

The samples were removed from -80°C storage and allowed to reach room temperature. One 1 x 1-inch section was cut from each sample using the sterile technique and placed, adhesive side down, into a glass jar containing 2 ml of extraction buffer (phosphate-buffered saline with 0.05% Tween20). A blank tape control was run in parallel for each type of tape to account for variation in background signal between sample types. Tapes were incubated with an extraction buffer at 100 rpm and 37˚C for one hour, followed by sonication for 10 minutes. After sonication, the solution was collected and centrifuged at 5000 g for five minutes at 4˚C. The supernatant was collected and placed into a new tube for BCA assay using a Micro BCA™ Protein Assay Kit following the manufacturer’s instructions (Thermo Fisher Cat# 23235).

Statistical Analysis for Study 1

Adhesion analysis was conducted using a mixed-model analysis of variance (ANOVA) of the raw data. BCA data analysis was conducted using a mixed-model ANOVA of the log-transformed values. For both endpoints, the sample was used as the fixed effect in the model, while the subject and the subject-by-sample interaction were included in the model as random effects. When needed, multiple comparison adjustments were made using the Tukey-Kramer adjustment. An alpha level of 0.01 was determined by using the Bonferroni correction to help control for type I errors across the five primary endpoints (α = 0.05/5 = 0.01). Analysis was conducted in SAS version 9.4 statistical analysis software (SAS Institute, Cary, NC).

Study 2

Study 2 was a balanced, complete block design with eight (8) samples on the volar forearms of 56 healthy human volunteers performed by an external contract laboratory (Dermico, Broomall, PA). The protocol was approved by the Allendale Institutional Review Board, and informed consent was obtained. There were eight types of tapes tested, including the same four tapes described in Study 1 and reported here (Table 1). From Study 2, the endpoints of interest that allowed us to quantitatively compare the four tapes were TEWL, inflammatory cytokines using IL-1a as a marker, and corneocytes removed by the tapes (percent area coverage). Controls consisted of skin sites without any tape applied and subjected to the same measurements as the test sites.

TEWL Method

A cyberDERM RG-1 Evaporimeter with Cortex DermaLab® TEWL probes was used to measure TEWL (cyberDERM, Broomall, PA). The recorded value is in g/m^2^*h and represents the water vapor coming through the skin. This is directly correlated with the skin barrier function. The subjects were acclimated for at least 30 minutes in an environmentally controlled room with their volar forearms exposed. A baseline measurement was taken for each test site and for a naïve control site. The tape samples were applied and pressed into place with a 4.5-pound roller (once forward and back) to ensure uniform contact. They were allowed to dwell for one minute and manually peeled using a “low and slow” method. Test sites were sequentially stripped 10 times, after which three TEWL readings were collected and averaged for each site. We have found especially when the skin is repeatedly stripped with adhesive tape that there can be marked differences in the degree of damage within the test site. For this reason, we configured the dual-probe evaporimeter with the probes joined side by side to simultaneously sample a larger region within the same test to obtain a more representative value for the change in TEWL due to stripping (Figure 4). The first and last tapes removed from each site were collected and stored adhesive side up in sterile trays at -20˚C until biological analysis. The remaining tapes were discarded. A final average value from a set of three TEWL measurements was then recorded for each test site. The difference in the TEWL values before and after stripping was used to evaluate the integrity of the skin barrier function.

Corneocyte Staining Method

The samples were removed from the -20°C storage and allowed to reach room temperature. A two-inch square area was cut from each sample and placed adhesive side down in staining solution (1% phloxine B in deionized water) for five minutes, followed by three to five washes in deionized water. When the deionized water remained clear after rinsing, the tape sample was placed adhesive side up and allowed to dry. Unworn tape samples were stained as controls for non-specific staining from backing materials. A Leica DVM6 digital microscope with the PlanAPO FOV 43.75 objective was used to collect images (Leica Microsystems, Wetzlar, Germany). Image-Pro Premier software was used to quantify the total percentage of positive staining from the surface area imaged for each sample after subtracting the control to remove non-specific staining.

IL-1a Method

After each test site was measured for TEWL as described above, a strip of 3M Transpore Surgical Tape (3M, St. Paul, MN), used for cytokine collection, was placed on the test site, allowed to dwell for one minute, and removed and saved for analysis. Cytokine tape collection was performed within 10 minutes after the tenth tape stripping and TEWL measurements were completed. Total protein was extracted from each cytokine collection tape as described previously, and the sample was analyzed using an automated enzyme-linked lectin assay (ELLA; BioTek, Winooski, VT) to quantify the level of proinflammatory protein IL-1a following the manufacturer’s instructions.

Statistical Analysis for Study 2

The changes in the data from baseline to the tenth successive tape removal were analyzed for each endpoint using a mixed-model ANOVA of the raw data. The sample, location, and sample-by-location interaction were used as the fixed effects in the model, while the subject was included in the model as the random effect. Data not normally distributed were rank- or log-transformed to help make reliable inferences. The model was reduced based on the significance of location and sample-by-location interaction terms. If the interaction term was significant, the endpoint was tested by location. When needed, multiple comparison adjustments were made using the Tukey-Kramer adjustment. In addition, Dunnett’s test was used when compared to the non-taped control site. A Bonferroni adjusted alpha of 0.0125 with Tukey’s test was used to adjust for multiple comparisons among the samples. Analysis was conducted in SAS version 9.4 statistical analysis software (SAS Institute, Cary, NC).

## Results

**Figure 1:**
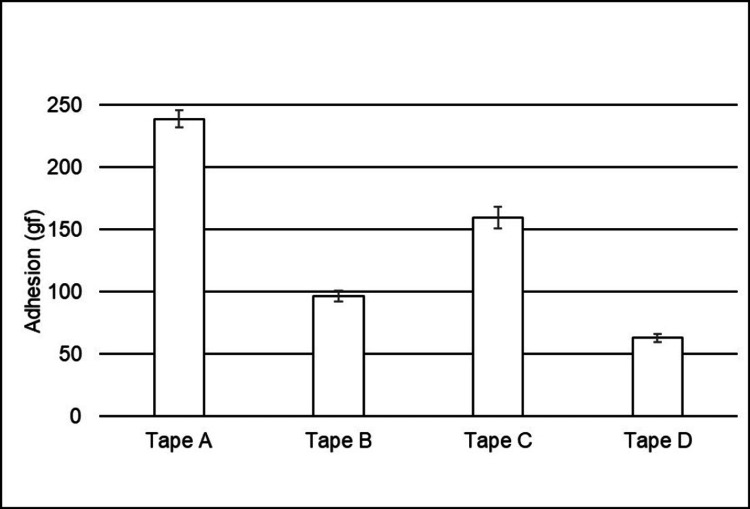
Mean adhesion forces (with standard errors) required to remove tapes after 24 hours of wear on the healthy human subjects. All tapes were statistically different from each other (p < 0.0001 for all comparisons).

**Figure 2:**
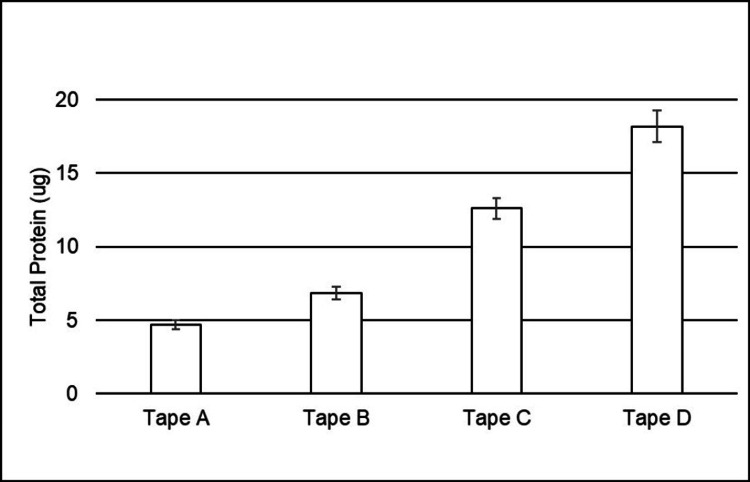
Quantitative analysis of the total protein left on tapes after 24 hours of wear time (BCA assay). Means and standard errors for each sample (n = 63, 60, 61, 61, respectively) were calculated from the individual values reported for all 32 subjects. All tapes were statistically different from one another (p < 0.0001 for all comparisons).

The healthy populations studied were considered comparable based on the inclusion and exclusion criteria. The results from Study 1 showed that Tape A adhered to the skin better than all other tapes tested (n = 58, p < 0.0001), as shown in Figure 1, but yet removed less total protein (BCA assay) from the skin than all other tapes (Figure 2). The testing of adhesion to the skin revealed that all four tapes were statistically different from one another, with Tape A having the highest adhesion levels and Tape D having the lowest levels (p < 0.001 for all comparisons). With respect to BCA, the testing revealed again that all four tapes were statistically different from one another, but this time, Tape A had the lowest BCA levels while Tape D had the highest levels (p < 0.0001 for all comparisons).

**Figure 3:**
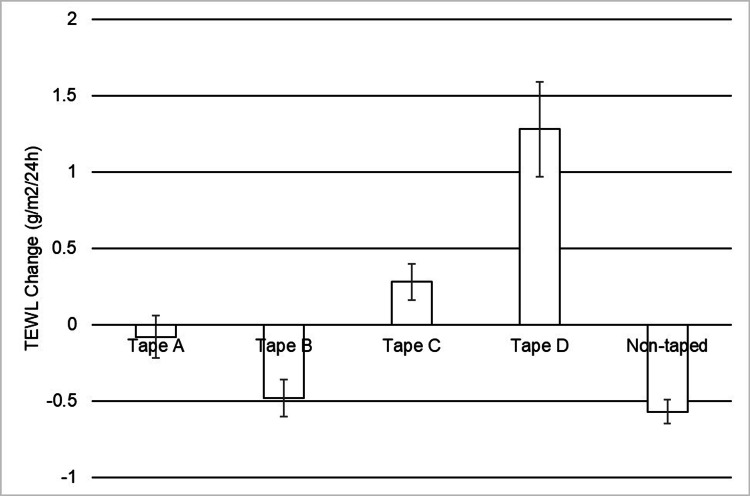
Change in TEWL measurement after repeated stripping of medical adhesives. The Y-axis indicates the difference in the TEWL measurement before and after tape stripping from each testing site. Each TEWL measurement represents the mean value (with standard error) taken from a set of three measurements at the same testing site at a given time. The TEWL difference shown in negative value is likely to be an artifact. The mean change in TEWL obtained from the sites from which Tapes A, B, and C were removed was found to be significantly less than that obtained from sites from which Tape D was removed (p < 0.003). Higher TEWL values were found for Tapes C and D compared to non-taped sites (p < 0.0005 for both comparisons). No statistical difference was observed between Tapes A or B and non-taped sites (p > 0.1221 for both comparisons).

The results from Study 2 showed that silicone-based medical adhesives do not alter the skin barrier. As shown in Figure 3, the mean change in TEWL from baseline revealed that there were no significant differences between the sites from which either Tape A or B was removed and the sites of non-taped control (p > 0.1221 for both comparisons). However, significantly elevated TEWL values were observed after serial applications and removals of Tapes C and D relative to non-taped sites (p < 0.0005 for both comparisons). In addition, the mean change in TEWL obtained from the sites from which Tapes A, B, and C were removed was found to be statistically lower than that obtained from the sites in which Tape D was removed (p < 0.0030 for all comparisons).

Corneocyte staining of the tape samples removed after repeated stripping in Study 2 showed a similar trend to the total protein results (BCA assay) from Study 1. After both the first tape stripping and the tenth tape stripping, Tapes A and B both had statistically lower mean percent corneocyte coverage than the acrylate-based tapes (p < 0.0001 for all comparisons).

**Figure 4:**
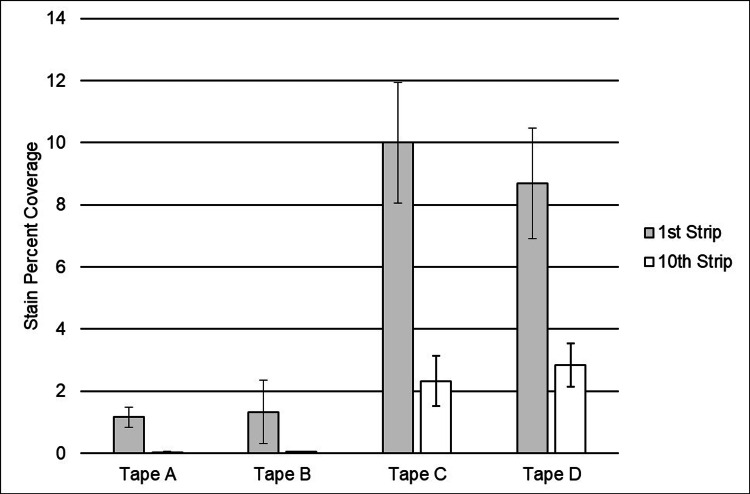
Results from the quantitative image analysis of corneocyte staining from tapes after the first and 10th strips. The same testing site underwent repeated stripping for each test article, with a one-minute dwell time in between each strip. The percentage of the total skin cell coverage over the approximately 1x1-inch square area was analyzed. Means and standard errors were calculated from the individual sample values reported from each of the 56 subjects. After the first tape stripping, Tapes A and B showed a statistically lower mean percent corneocyte coverage than Tapes C and D (p < 0.0001). After the tenth tape stripping, Tapes A and B showed a statistically lower mean percent corneocyte coverage than Tapes C and D (p < 0.0001).

For tapes collected from the first stripping, there was a larger percentage of the surface area covered by corneocytes with acrylate-based tapes compared to the silicone-based tapes (Figure 4). After the tenth stripping, the surface area covered by cells was nearly zero for Tapes A and B (silicone adhesives). However, corneocytes were still visible for Tapes C and D (acrylate adhesives), although in a decreased amount compared to the first stripping.

**Figure 5:**
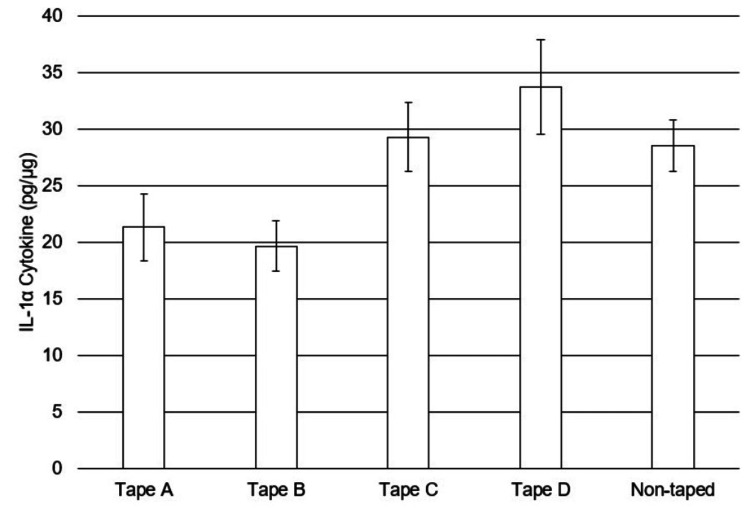
Quantification of the secreted IL-1a after repeated stripping. The amount of IL-1a from each site was normalized to the amount of total protein detected from the collection tape. The baseline quantity of IL-1a detected from the non-taped control site was also shown here. The Y-axis represents the mean values and standard errors from the 56 test subjects. Since the results from each sample comparison were confounded by location, no overall statistical conclusions could be drawn.

Collection tapes were removed and analyzed for the quantification of secreted IL-1alpha (used as a marker of inflammation). Data (Figure 5) were analyzed via two models due to the non-taped site being at a fixed location on the middle volar forearm (tape locations were randomized). Statistical analysis using the first model included the non-taped site compared to the taped sites and showed a statistically higher mean cytokine value for non-taped sites than for skin sites under the two silicone adhesive tapes (p < 0.0001). No other comparisons were found to be statistically significant. Statistical analysis with the second model excluded the non-taped skin site and showed a statistically significant sample by the location effect (p = 0.0005), which means that any overall sample comparisons are confounded by location on the forearm. Therefore, results had to be analyzed by location. Statistically significant observations between tapes were not consistent across all locations on the forearm, so no overall conclusions could be drawn.

## Discussion

In this paper, we have compared four medical tapes for their adhesion to the skin, amount of total protein and corneocytes removed, amount of IL-1a production induced, and effect on the TEWL of the skin. We included two tapes with acrylate-based adhesives and two with silicone-based adhesives across two studies.

It is generally believed that since the removal of silicone-based adhesives is less impactful, they may not adhere as well to the skin. For example, Klode et al. [19] tested adhesion in wound dressings and compared multiple types of adhesives (acrylate, silicone, hydrocolloid, and polyurethane). They reported that overall, silicone-based adhesives required the lowest amount of energy to remove and that there was a significant correlation between adhesion and pain intensity. Conversely, other studies have found a poor correlation between the peel force and skin damage or discomfort [20-22]. To evaluate the relative performance of silicone- and acrylate-based adhesive tapes, our study assessed adhesion to the skin by measuring the removal force after 24 hours of wear. The results (Figure 1) show that Tape A (silicone-based) had higher adhesion than all other tapes and Tape B (also silicone-based) had higher adhesion than Tape D (acrylate-based), indicating that a silicone adhesive can adhere better than acrylate-based adhesives and that all silicone adhesives are not equivalent. These data suggest superior performance in securement for Tape A.

The BCA assay is a common method for quantifying proteins in a solution. After extracting proteins from removed tape samples, the assay provides a way to evaluate the extent of skin stripping caused by the removal of an adhesive device by measuring the amount of protein in the stratum corneum removed [7]. The extraction technique used in this study allows quantification of both soluble and insoluble proteins on the tape samples that had been worn on the backs of human subjects for 24 hours. Another approach to evaluate the physical impact of skin stripping, and therefore the risk for MARSIs, is the quantification of the SC layer being removed by tapes. This technique provides an orthogonal approach to assessing skin stripping that can be used to support the data from BCA assay analysis. Interestingly, the higher adhesion we observed in our study did not correlate with the amount of total protein removed, since Tape A contained less total protein, on average, than all other tapes (p < 0.0001), ranging from 31% less compared to Tape B to up to 74% less than Tape D (Figure 2). As expected, the measurement of corneocytes removed followed the same trend as the total protein results (Figures 2 and 4 both show less material removed by silicone tapes than by acrylate tapes), since both tests evaluate the extent of skin stripping caused by the removal of the adhesive device. Corneocyte staining data from the repeated stripping study on the healthy human subjects' volar forearms corroborated the BCA data collected in the 24-hour wear study. Staining and image analysis showed significantly less protein removed by both silicone adhesive tapes compared to the acrylate adhesive tapes after both one and 10 strippings (p < 0.0001; Figure 4).

A previous study conducted on wound dressings measured the peel force needed for removal and the amount of stratum corneum removed and found that the relationship between the degree of damage and peel force is not clear-cut. The selection of dressings tested included one with a silicone adhesive, which required more force to peel off than a dressing with a hydrocolloid adhesive, and yet removed less stratum corneum. The authors hypothesized that factors other than peel force must also determine the level of damage to the skin surface [20]. Tokumura et al. conducted a study on tapes comparing acrylic adhesives in different environmental conditions and also found that a higher peel force did not correlate with a higher number of corneocytes stripped [15]. In addition, they noted that the number of corneocytes removed decreased with additional stripping iterations, in alignment with our observations (Figure 4). A possible explanation for this is that corneocytes at the surface are dead and not bound strongly to each other and therefore lift more easily. After repeatedly peeling the surface, corneocytes from deeper layers, which have stronger intercellular binding, do not come off as easily [15]. Our adhesion and corneocyte results therefore confirm observations made by others.

TEWL measures the amount of water per area and time that evaporates from the surface of the skin to provide an indication of moisture barrier function [23]. It is important to note that it is the relative change in TEWL over time compared to baseline measurement that provides an indication of compromised moisture barrier function rather than the absolute TEWL values themselves. TEWL fluctuation depends on both intrinsic and extrinsic factors. In our study, these fluctuations were accounted for by assessing adjacent naïve control sites on the volar forearms of all subjects. The small shifts in TEWL under taped sites versus non-taped sites were not enough to cause adverse events in the healthy human subject study. However, the trends suggest that silicone-based adhesives do not negatively impact moisture barrier function in healthy populations. No statistical differences in TEWL were observed between naïve sites (control) and sites that had 10 strips of Tape A sequentially applied and removed (Figure 3). Tape B (silicone-based adhesive) also showed a negligible change in TEWL. This is not unexpected as minimal changes in TEWL after repeated stripping of silicone adhesive products have been previously documented [8,10]. Thus, Tapes A and B do not significantly disrupt the stratum corneum barrier and offer a lower risk of compromised barrier function from physical insult when compared to the two acrylate adhesive tapes assessed. Unlike what was observed with the silicone-based adhesives, changes in TEWL were recorded from the acrylate-based adhesives post skin stripping (Tapes C and D). Such changes were also reported by others [9,10,15]. In our study, Tape D caused the largest disruption in the skin barrier and adhered the least. It is possible that the type of backing has an impact; a plastic backing likely traps more moisture under the tape over time, which could interfere with adhesion. Our data suggest that silicone-based adhesive products are a better choice than their acrylate-based counterparts to protect the skin barrier when treating patients with fragile skin conditions.

Using the same extraction technique described for total protein analysis, other analytical techniques, such as immunoassays, can be used to look at inflammatory markers. For example, Perkins et al. [24] specifically described the use of a tape method to analyze inflammatory molecules including IL-1a to compare diseased and normal scalps. We applied the same principle to compare non-taped skin and skin subjected to repeated tape applications and removals to gauge the level of irritation caused by the various tapes. Our cytokine analysis, although confounded by a significant location effect, appears to show, at least directionally, that the two silicone adhesives were gentler to the skin than the acrylate adhesives (Figure 5), even though the differences were not consistent across the location on the forearm.

The lack of difference between the taped and non-taped sites could result from, in part, low MARSI induction, if any, from taped sites. This outcome was not unexpected as the design of the clinical study did not aim to artificially induce MARSIs by aggressive stripping. In addition, medical tapes selected in this study are commonly used in clinical settings with little to no reports of MARSIs or significant inflammation. Despite the findings, there were several test sites where silicone-based tapes displayed significantly lower IL-1a results than acrylate tapes, but unfortunately, there was no consistency across all locations on the forearm, and no conclusions could be drawn on this endpoint. Overall and despite this variability, the general lack of IL-1a induction observed suggests that stripping the skin 10 times in a row is likely not enough to induce detectable inflammation.

The limitations of this study include the small number of tapes tested and the fact that the subjects were healthy volunteers, not reflecting fragile skin seen in typical patients. However, testing for skin damage with repeated applications and removals could not be ethically done in fragile skin patients, and healthy volunteers provide a more clinically relevant model as opposed to using a bench method (such as adhesion to steel) or animal model. They also provide a more consistent and uniform baseline (more similar skin properties and less variability than fragile patients). Another limitation is that in this study, we did not test adhesion to devices, such as various tubing materials.

## Conclusions

The human skin data presented here clearly indicate that different levels of risk for damage and disruption to skin exist between medical adhesive tapes. The silicone adhesive tapes were less disruptive to the skin barrier than either of the acrylate adhesive tapes (as measured by TEWL, BCA assay, and corneocyte staining). The differences observed were small given the fact that the tapes used are not considered very aggressive and our testing was done on healthy volunteers and not in patients with fragile skin. In addition to having a minimal relative impact on skin, Tape A consistently performed better in efficacy (as measured by adhesion to the skin) than all other tapes studied. It is our hope that clinicians can use these data to improve their understanding of how medical adhesive products are linked to skin injuries and that researchers can use these methods to guide the development of products that will mitigate the risk of MARSIs and potential subsequent HAIs, ultimately leading to better patient outcomes.
